# Acceptability of the distribution of DMPA-SC by community health workers among acceptors in the rural province of Lualaba in the Democratic Republic of the Congo: a pilot study^☆^

**DOI:** 10.1016/j.contraception.2018.08.004

**Published:** 2018-11

**Authors:** Albert Mwembo, Rebecca Emel, Tesky Koba, Jacqueline Bapura Sankoko, Aben Ngay, Rianne Gay, Jane T. Bertrand

**Affiliations:** a École de Santé Publique de Lubumbashi, University of Lubumbashi, Lubumbashi, Democratic Republic of the Congo; b Global Health Management and Policy, Tulane University School of Public Health and Tropical Medicine, 1440 Canal St, Suite 1900, New Orleans, LA 70112, USA; c l'Univers Santé Plus, Democratic Republic of the Congo; d Pathfinder International-Democratic Republic of the Congo; e Pathfinder International-Democratic Republic of the Congo (at the time of the study)

**Keywords:** DMPA-SC, Sayana® press, Community health workers (CHWs), Acceptability, Rural Africa

## Abstract

**Objectives:**

The objective of this research is to assess the acceptability of the provision of subcutaneously administered depo medroxyprogesterone acetate (DMPA-SC) by nonclinically trained community health workers (CHWs) among acceptors in the rural province of Lualaba in the Democratic Republic of the Congo (DRC).

**Study design:**

In 2017, 34 CHWs received training in provision of DMPA-SC. Among other methods, DMPA-SC by CHWs was offered during household visits and at community outreach events. The initial survey included questions on acceptors' demographic characteristics, contraceptive use history and experience with provision of DMPA-SC by a CHW. The follow-up included questions about side effects experienced and continuation of DMPA-SC by a CHW.

**Results:**

Seventy-four percent of initial acceptors of DMPA-SC (*N*=252) were first-time contraception users. Almost all (96.0%) felt very comfortable with a CHW performing the injection rather than a physician or nurse, and 97.6% perceived that the CHW was very comfortable performing the injection. A total of 239 women were interviewed at follow-up. Most expressed satisfaction with the method despite some side effects experienced. Almost all acceptors (97.9%) were satisfied with the information provided by CHWs, and 93.8% were satisfied with the overall service. Most (96.4%) would choose to continue receiving DMPA-SC by a CHW rather than in a health clinic, and 95.2% would recommend DMPA-SC by a CHW to a friend.

**Conclusions:**

Overall, administration of DMPA-SC by CHWs is acceptable to users in Lualaba. DMPA-SC can be safely provided within the community after proper training.

**Implications:**

This study validates the use of CHWs (without clinical training) to provide DMPA-SC in a rural sub-Saharan African setting. It also represents an important step in obtaining official MOH authorization for the scale-up of this mechanism of distribution to other underserved regions in the DRC.

## 1 Introduction

The use of modern contraception has been increasing across sub-Saharan Africa, but access and method availability are still limited in rural and hard-to-reach areas. Community health worker (CHW) programs, when appropriately designed, can reduce inequalities in access to services by bringing the products and information to people in their communities rather than requiring them to visit a health clinic. CHWs have been providing a variety of nonclinical contraceptive methods, such as oral contraceptives, condoms and spermicides, since the 1980s, but these types of methods may not meet the needs of all women [1].

Injectables are one of the most popular modern contraceptive methods in sub-Saharan Africa, but in many countries, their provision is restricted to nurses or physicians [2], [3]. As a result, access to injectables is limited, especially among women in geographically isolated areas where health facilities are sparse. Task-shifting, or the delegation of tasks and responsibilities to different cadres of health workers, is a widely accepted means of addressing workforce shortages, improving geographic equity and increasing cost-effectiveness [4]. Training lower cadres of health workers — such as CHWs — to provide injectable contraceptives can expand access and reduce unmet need in underserved areas without adding excessive strain on the health sector [5].

Most of the literature to date on community-based provision of injectables has focused on the 3-month intramuscular administrated depo medroxyprogesterone acetate (DMPA-IM), which comes in a vial and requires a separate syringe for administration [6]. In 2013, a new formulation of DMPA-IM was accepted [6]. This newer formula, subcutaneously administered depot medroxyprogesterone acetate (DMPA-SC), can be administered through a prefilled Uniject™ injection system, which does not require separate vials or syringes. This formulation has great potential for distribution in community settings and may therefore increase modern contraceptive use worldwide, especially among women in underserved areas [6].

Pilot studies in Senegal and Uganda suggest that its all-in-one design makes DMPA-SC easier and faster to prepare and administer, and participants found it less painful than DMPA-IM [7], [8]. These studies revealed that 80% of participants in Senegal and 84% of participants in Uganda would chose DMPA-SC over DMPA-IM [7]. A 2015 pilot study in Kinshasa, Democratic Republic of the Congo (DRC), which used medical and nursing students as community-based distributors, also found a high level of acceptability among DMPA-SC users. Approximately 9 in 10 DMPA-SC acceptors reported satisfaction with the services they received and would recommend the method to a friend [9]. The success of the Kinshasa pilot has opened the door for new task-shifting initiatives in the DRC, which include the community-based intervention detailed in this pilot, as well as using medical and nursing students to teach women self-injection of DMPA-SC [10].

According to the 2013–14 Demographic and Health Survey, only 4.6% of married women in rural DRC were using a modern contraceptive, whereas over one quarter (27.3%) had an unmet need for contraception [11]. In rural DRC, facilities offering quality FP services are sparse, and even when contraceptives are physically available, barriers related to cultural norms and cost may limit access. With 57% of its 81.5 million inhabitants living in rural areas, FP services in the DRC are out of reach for a vast portion of the population, resulting in one of the highest fertility rates in the world [12].

Injectables are the second most popular method in the DRC [11]. Provision of injectables by CHWs in the form of DMPA-SC is expected to improve access for women in underserved areas of the country, thus increasing modern contraceptive use in this population. This service delivery mechanism may also reduce some of the burden on the health care system. CHWs operate on a volunteer basis, resulting in high personnel savings for each dose of DMPA-SC provided by a CHW rather than a nurse or physician. Additionally, when CHWs provide DMPA-SC, nurses and physicians can focus their time on the more clinically invasive procedures such as insertion and removal of implants. This pilot assesses the acceptability of the provision of DMPA-SC by nonclinically trained CHWs among acceptors in a rural region of the DRC.

## 2 Materials and methods

### 2.1 Pilot intervention

In 2014, the United States Agency for International Development's Evidence to Action for Strengthened Family Planning and Reproductive Health Services for Women and Girls Project (E2A) was enacted in the DRC [13]. Led by Pathfinder International, working in collaboration with the Ministry of Health (MOH), E2A aims to “improve the quality of integrated community-based family planning and maternal, newborn and child health services” [13]. The pilot intervention detailed in this paper is an extension of E2A's work in the DRC.

The E2A project covers 51 health areas in 15 health zones (HZs) in 3 provinces in the DRC: Lualaba, Kasai Central and Lomami [13]. Central to the E2A project is the cadre of CHWs (known locally as “RECOS” for *relais communautaires*) who receive direct support from local nurses and health authorities [13]. Under this project, CHWs conduct household visits and participate in community outreach events (*Stratégie Avancée)* to provide FP information and counseling, and a range of nonclinical contraceptive methods including male and female condoms, pills and Cycle Beads® [13].

Under E2A, a health team including a nurse from the fixed facility and one or more CHWs arrive in a central location once a month to provide health services. During each *Stratégie Avancée*, CHWs hold awareness sessions describing the full range of contraceptive methods. If a woman chooses a method CHWs are authorized to provide (pills, condoms or CycleBeads®), she participates in an individual counseling session to determine her eligibility for that method before receiving it from a CHW. Those who select a method requiring clinician administration (implant, intrauterine device or injectable) are directed to a registered nurse to administer the method. Under the E2A program, nurses provide DMPA-IM to clients interested in receiving an injectable. This pilot study expands the E2A project by training CHWs to provide DMPA-SC during *Stratégie Avancée* and home visits, freeing up the nurse to tend to other healthcare delivery services.

CHWs who were already trained in the E2A project and met the selection criteria^1^ were invited to participate in the training of DMPA-SC provision. The training, which ranged from 5 to 7 days depending on the CHW's knowledge and skill level, included a brief refresher of other contraceptive methods but focused on teaching CHWs to inject DMPA-SC and counsel on its side effects. It also included training with a standardized eligibility checklist specific for each available method. The checklist included pregnancy screening questions, but in some cases, rapid pregnancy tests (BetaHCG) were used to confirm pregnancy status. After demonstrating competence using anatomical models, CHWs also participated in a field exercise that included close supervision by their trainers in a real-world setting.

Upon successful completion of the training, CHWs were supplied with DMPA-SC (in the form of Sayana® Press, manufactured by Pfizer, Inc., and delivered through the Uniject™ injection system) as a method they were authorized to provide in the community, both during the *Stratégie Avancée* and door-to-door. A total of 34 CHWs participated in the pilot. The average age of participating CHWs was 42.2 years, 52.9% (*n*=18) were female, almost three quarters were married (*n*=25), and all but one CHW had children (mean number: 5.8 children) [14].

The province of Lualaba was deliberately selected for this pilot due to civil unrest in the other provinces. Two HZs in Lualaba (Fungurume and Bunkeya) were selected according to three criteria: their rurality, relatively dense populations and accessibility. Table 1 provides demographic characteristics of the province of Lualaba compared to the DRC as a whole. Women in Lualaba are less educated, less likely to be using a modern method and are more likely to have an unmet need for limiting than women across the country.

**Table 1:** Weighted demographic characteristics of women ages 15–49 in the Province of Lualaba compared to the Democratic Republic of the Congo as a whole^a^

Characteristics	DRC *N*=18,827		Lualaba *N*=459		p value^b^
	*n*	%	*n*	%	
Age (years)					
Mean	28.1	--	28.2	--	
Education					
None	2903	15.4	110	24.0	<.01
Primary	6949	36.9	217	47.3	<.01
Secondary	8287	44.0	129	28.1	<.01
Higher	688	3.7	3	0.7	<.01
Contraceptive use					
No method	15,201	80.7	407	88.7	<.01
Folkloric method	169	0.9	16	3.5	<.01
Traditional method	1927	10.2	19	4.1	<.01
Modern method	1529	8.1	17	3.7	<.01
Any living children					
Yes	13,909	73.9	343	74.7	
# of living children					
Mean	3.7	--	3.6	--	
Unmet need					
For spacing	2933	15.6	70	15.3	
For limiting	968	5.1	45	9.8	<.01

a Data are from the 2013–14 Demographic and Health Survey conducted in the Democratic Republic of the Congo.

b Two-sample *t* test.

Women who selected DMPA-SC by a CHW were invited to participate in a short survey about their experience. Upon agreeing, these acceptors were enrolled into the study. The sample includes a total of 252 women.

### 2.2 Data collection

#### 2.2.1 Initial acceptor survey (June–July 2017)

Ten female interviewers received training on the questionnaire and survey protocol. The interview team accompanied CHWs to the *Stratégie Avancée* to enroll clients who selected and received DMPA-SC. Interviews followed an approved script, in which the study objectives were explained to the acceptor and informed consent was obtained. The initial survey included questions on acceptors' demographic characteristics, contraceptive use history, and experience with counseling and injection of DMPA-SC by a CHW. In cases where DMPA-SC was provided during a home visit outside of the *Stratégie Avancée*, the interview took place following the same procedure provided an interviewer was available nearby to enroll the clients who agreed to participate.

#### 2.2.2 Three-month follow-up survey of acceptors (September–October 2017)

Approximately 12 weeks after the initial injection, the research team contacted each eligible respondent who received DMPA-SC, took part in the initial interview and agreed to be contacted for the follow-up survey. The follow-up included questions about side effects experienced and continuation of DMPA-SC by a CHW.

All survey data were collected using encrypted smartphones programmed with the OpenDataKit (ODK) application. Transmission of the data through ODK to the online server, FormHub, provided the study personnel with immediate access to the data. Respondents received no compensation for their participation in this study. The survey data were analyzed using STATA/SE 13.

Data collection for this research was approved by Tulane University Institutional Review Board (16-#911338) as well as by the Ethics Committee of the Kinshasa School of Public Health (#ESP/CE/071/2016).

## 3 Results

### 3.1 Initial acceptor survey

The average age of DMPA-SC acceptors was 27.7 years. Only 30.2% had some secondary education or higher. Over half were employed (56.0%), but most of these women worked informally (working for family members or receiving in-kind payment). The majority were married or lived in union (79.0%), and nearly all (97.6%) had children (mean number: 4.4).

Almost three quarters of all acceptors (74.2%) had never used a contraceptive method prior to this pilot. Among those who had used a method before, just over half (55.4%) had previously used an injectable. Reasons for selecting DMPA-SC (*n*=252) over another method included effectiveness (94.4%), fewer health risks (11.1%) and convenience/ease of use (7.6%) (multiple responses possible). Table 2 shows demographic characteristics of initial acceptors by previous use of contraception.

**Table 2:** Characteristics of initial acceptors of DMPA-SC by previous use of contraception

Characteristics	Initial DMPA-SC Acceptors *N*=252				
	First-time FP users *n*=187		Continuing FP users *n*=65		p value^a^
Age (years)	**n**	**%**	**n**	**%**	
Mean	26.8	--	30.4	--	<.01
Marital status					
Not in union	40	21.4	13	20.0	
Married/in union	147	78.6	52	80.0	
Education					
None	50	26.7	9	13.8	<.05
Primary	86	46.0	30	46.2	
Secondary or higher	51	27.3	25	38.5	
No response	0	0.0	1	1.5	
Any living children					
Yes	182	97.3	64	98.5	
Number of living children					
Mean	4.1	--	5.3	--	<.01

a Two-sample *t* test.

Although many women were very anxious before receiving the injection (42.1%), most reported the injection as painless or only slightly painful (96.8%). Almost all (96.0%) felt very comfortable with a CHW performing the injection rather than a physician or nurse, and 97.6% perceived that the CHW was very comfortable performing the injection. Acceptors also perceived CHWs as being very comfortable explaining the method and its side effects (97.2%). Most of the initial acceptors (64.4%) received their injection from a CHW at home, and almost one third (28.0%) received their injection at a *Stratégie Avancée*. The remaining women received their injection either at the CHW's house or outside of a friend or family member's house.

Table 3 shows demographic characteristics of women who received their first injection at home versus at a *Stratégie Avancée*. Women who received DMPA-SC at home were more likely to be unmarried, be employed, have fewer children and have a partner or husband who agrees with her FP use compared to women who received DMPA-SC at a *Stratégie Avancée*.

**Table 3:** Characteristics of acceptors of DMPA-SC based on location of service provision

Characteristics	Home visit *N*=154		*Stratégie Avancée* *N*=67		p value^a^
	*n*	%	*n*	%	
Age (years)					
Mean	28.3	…	29.4	…	
Marital status					
Married/in union	124	80.5	58	86.6	
Divorced	12	7.8	3	4.5	
Widow	1	0.6	2	3.0	
Never married	17	11.0	4	6.0	<.05
Education					
Primary	95	61.7	41	61.2	
Secondary	47	30.5	16	23.9	
Other	12	7.8	10	14.9	
Employment					
Cash job	28	18.2	5	7.5	<.05
In kind job	38	24.7	28	41.8	<.01
No job	88	57.1	34	50.7	
First time user					
Yes	107	69.8	49	73.1	
Any living children					
Yes	153	99.4	66	98.5	
Number living children					
Mean	4.3	…	5.0	…	<.05
Does your husband agree with your FP use?					
Agrees	74	48.1	47	70.1	<.01
Disagrees	49	31.8	16	23.9	
Don't know	31	20.1	4	6.0	<.01

a Two-sample *t* test.

### 3.2 Three-month follow-up survey of acceptors

Of the 252 initial acceptors, 239 (94.8%) were interviewed 12 weeks later. Among these women, 40.6% experienced side effects after their first DMPA-SC injection. Among those reporting side effects (*n*=97), the most frequently reported included irregular periods (48.5%), no period (38.1%), abdominal pain (30.9%), heavy bleeding (17.5%%) and headaches (13.4%) (multiple responses possible). Among the 17 previous users of DMPA-IM, 64.7% reported similar side effects, and 23.5% experienced fewer side effects with DMPA-SC.

At the 3-month follow-up, 5 of the 239 respondents (2.1%) indicated that they became pregnant since receiving DMPA-SC. Follow-up was conducted with 3 of these cases. Two indicated that they were aware of their condition but did not want to report this information to the CHW in the hopes that DMPA-SC would have an abortive effect. The third woman was unaware of her pregnancy at the initial interview due to her irregular menstrual cycle.

Most (92.1%) of the women interviewed at follow-up (*n*=239) either chose to have a second injection or planned to have a second injection of DMPA-SC. At the time of the follow-up interview, some women did not yet have their second appointment with a CHW. Of those women (*n*=28), just over one third planned to choose a second injection of DMPA-SC. Reasons for intending to continue use of DMPA-SC included ease of use, few side effects and convenience due to not having to go to a facility. All women interviewed at follow-up who had a second appointment with a CHW (87.9%) chose to have a second injection of DMPA-SC. Almost all (98.6%) of those who did not experience a side effect either received a second injection of DMAP-SC or planned to receive a second injection.

Of all 239 women who were interviewed at follow-up, 92.1% would recommend DMPA-SC as a method to a friend or family member who wanted to delay or avoid pregnancy, and 94.6% would choose to continue receiving DMPA-SC in the community by a CHW rather than in a health facility. Almost all acceptors (97.9%) were satisfied with the information provided by CHWs, and 93.8% were satisfied with the overall service. Table 4 shows initial levels of satisfaction with DMPA-SC by a CHW by method continuation. Those who had a second dose of DMPA-SC or planned to have a second dose were more likely to be very satisfied with the information and advice received from the CHW and were more likely to strongly recommend DMPA-SC by a CHW to a friend than those who did not plan to continue DMPA-SC.

**Table 4:** Satisfaction with DMPA-SC by a CHW by method continuation

Initial satisfaction with CHW	3-month follow-up *N*=239				
	Continued or plans to continue DMPA-SC *n*=220		Does not plan to continue DMPA-SC *n*=18^a^		p value^b^
	n	%	n	%	
How comfortable were you having CHW inject you rather than a doctor/nurse?					
Very comfortable	187	85.0	14	77.8	
Somewhat comfortable	32	14.5	4	22.2	
Uncomfortable	0	0.0	0	0.0	
Level of satisfaction regarding information/advice from CHW					
Very satisfied	163	74.1	10	55.6	
Somewhat satisfied	55	25.0	6	33.3	
Unsatisfied	1	0.5	1	5.6	<.05
Level of satisfaction regarding overall satisfaction with service provided by CHW					
Very satisfied	168	76.4	11	61.1	
Somewhat satisfied	50	22.7	6	33.3	
Unsatisfied	1	0.5	0	0.0	
Would you recommend DMPA-SC by a CHW to a friend?					
Strongly recommend	144	65.5	7	38.9	<.05
Recommend	72	32.7	3	16.7	
Do not recommend	3	1.4	6	33.3	<.01
Don't know	0	0.0	2	11.1	<.01

a Includes three “Does not know” values. Continuation totals will not equal 239; there is 1 missing value (see Table 3). Totals may not add up to 220/18; missing values were not included in the table.

b Two-sample *t* tests.

Table 5 shows initial levels of satisfaction by location of initial service provision. Those who received DMPA-SC at home were more likely to be very satisfied by the overall service provided by the CHW and were more likely to recommend or strongly recommend DMPA-SC by a CHW to a friend than those who received DMPA-SC at a *Stratégie Avancée*.

**Table 5:** Satisfaction with DMPA-SC by a CHW by location of initial service provision with two-sample test of proportions^a^

Initial satisfaction with CHW	3-month follow-up *N*=221^a^				
	Received first dose at home *n*=154		Received first dose at *Stratégie Avancée* *n*=67		p value^b^
	n	%	n	%	
How comfortable were you having CHW inject you rather than a doctor/nurse?					
Very comfortable	134	87.0	55	82.1	
Somewhat comfortable	20	13.0	12	17.9	
Uncomfortable	0	0.0	0	0.0	
Level of satisfaction regarding information/advice from CHW					
Very satisfied	121	78.6	46	68.7	
Somewhat satisfied	32	20.8	20	29.9	
Unsatisfied	1	0.6	0	0.0	
Missing	0	0.0	1	1.5	
Level of satisfaction regarding overall satisfaction with service provided by CHW					
Very satisfied	125	81.2	47	70.1	
Somewhat satisfied	29	18.8	18	26.9	
Unsatisfied	0	0.0	1	1.5	
Missing	0	0.0	1	1.5	
Would you recommend DMPA-SC by a CHW to a friend?					
Strongly recommend	95	61.7	52	77.6	<.05
Recommend	52	33.8	13	19.4	<.05
Do not recommend	5	3.2	2	3.0	
Don't know	2	1.3	0	0.0	
Would you be willing to continue receiving DMPA-SC by a CHW?					
Yes	148	96.1	63	94.0	
No	6	3.9	3	4.5	
Don't know	0	0.0	1	1.5	

a Total includes only those who received initial injection at home or at a *Stratégie Avancée.* “Other” category not included.

b Two-sample *t* test.

**Fig. 1:**
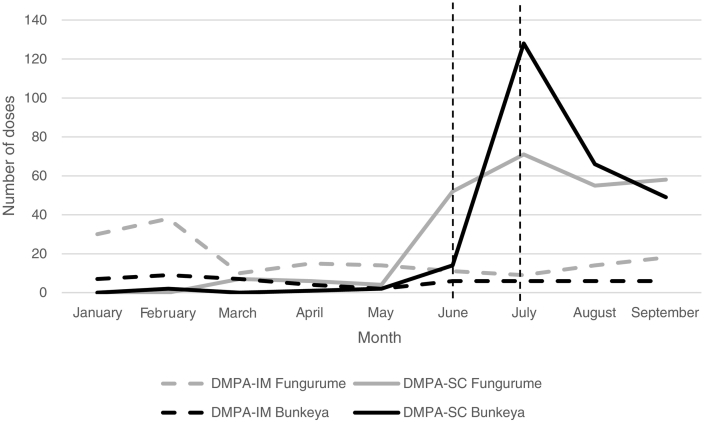
Doses of DMPA-IM and DMPA-SC provided during *Stratégie Avancée* by month.* *The initial phase of this pilot took place from June to July, indicated by the vertical dashed lines.

Fig. 1 shows the number of doses of DMPA-IM and DMPA-SC provided at the *Stratégie Avancée* by HZ. Doses of DMPA-SC provided in Fungurume increased from 4 to 71 during the initial phase of the pilot (June to July) and from 2 to 128 during the same period in Bunkeya.

## 4 Discussion

Use of CHWs to perform injections of DMPA-SC and provide counseling on contraceptive methods and their side effects is acceptable among users in Lualaba, given the overwhelmingly positive reaction in this pilot study. Almost all women who received the injections from a CHW indicated a high level of satisfaction with the information provided and the services received. The proportion of acceptors who received a second dose of DMPA-SC is similar to that found in Bertrand et al. (94.0%) [9]. This observation reflects high user acceptance of a community-based approach to distribution of DMA-SC in two different contexts in the DRC.

Consistent with existing literature on community distribution of injectables, acceptors in this pilot favored the mechanism of delivery of DMPA-SC by CHWs in the community. At follow-up, most women reported they would choose to continue receiving injections in the community by a CHW rather than at a health center.

Distribution of DMPA-SC by CHWs not only increased the number of injectables provided from the period prior to the pilot (see Fig. 1) but may have also improved access to injectable methods among women who were unable to attend a clinic or *Stratégie Avancée*. Most women in this pilot received DMPA-SC in their home rather than at a *Stratégie Avancée*. A large portion of these women were first-time contraceptive users, and they preferred home visits due to the easy/convenient location, lower cost for travel, lack of waiting/lines and availability of their preferred method. Women who received DMPA-SC from home expressed more satisfaction with the services received and were more enthusiastic about recommending the distribution mechanism than women who attended the *Stratégie Avancée.* CHWs have the unique ability to bring FP services to women in their community, and when these services include highly desired methods such as injectables, the potential impact on modern contraceptive use and unmet need is great.

One disturbing result from this pilot is the five pregnancies reported after receiving DMPA-SC. Under the pilot, pregnancy screening questions were included as part of the eligibility criteria, and in more complicated cases, rapid pregnancy tests were administered. Despite this standardized procedure, not all pregnancies were captured. This finding underscores the need for a more robust screening process or mandatory rapid pregnancy tests for all women who choose this method.

### 4.1 Limitations

Ideally, we would have interviewed the first 250 acceptors of DMPA-SC who enrolled in the program to reduce selectivity bias. However, because it was not always possible to have an interviewer available, especially outside the *Stratégie Avancée*, we attempted to interview every acceptor that selected DMPA-SC provided by a CHW; the result was a convenience sample.

At the 3-month follow-up, a total of 13 women (5.2%) were lost-to-follow-up. This situation is a predictable risk in follow-up or cohort studies and can be due to several reasons. For our case, many clients had gone to work in their fields at this time. However, this proportion of loss-to-follow-up is common in these types of studies and does not alter the results of the work.

Baseline data prior to the start of this pilot were lacking. Conclusions from this pilot may have been strengthened if service statistics for nurses and CHWs at the *Stratégie Avancée* were reported separately and if service statistics for home visits were captured. As is, we cannot tell the extent to which nurse productivity increased with the presence of CHWs, nor can we determine the number of women who received DMPA-SC from a CHW but refused to be interviewed. Because they were not the main priority of the pilot, we know little about CHW home visits — when and where they took place, how the CHWs selected the homes they visited and how many women received DMPA-SC at home but refused to be interviewed. This information may have provided more insight on the unique characteristics of these acceptors and helped to quantify the potential reach of a CHW program in this setting.

### 4.2 Implications and next steps

This pilot was intended to assess the acceptability of CHW provision of DMPA-SC at scheduled *Stratégie Avancée* days. The number of acceptors who received DMPA-SC at home was a surprising finding. These acceptors expressed higher levels of satisfaction and were more enthusiastic about recommending the service mechanism to a friend or family member than acceptors who received the method from a *Stratégie Avancée.* Moving forward with the scale-up of this pilot, home visits will become a larger focus. To remain active, CHWs will be required to attend only one *Stratégie Avancée* per quarter (with the option to attend more) to report service statistics, restock methods and check in with a supervisor. *Stratégie Avancée* days will continue with rotating locations to reach the maximum number of women.

Given the five undetected pregnancies, the screening checklist used in this pilot to determine eligibility for a woman's preferred method must be revised before scale-up. The research team may also opt to administer rapid pregnancy tests for each woman before providing hormonal methods.

Results from the companion article *Task-shifting the provision of DMPA-SC in the DR Congo: perspectives from different groups of providers* showed that CHWs were not satisfied with the compensation received [14]. Under this pilot, CHWs operated on a volunteer basis. In the scale-up of this program, contraceptives will be offered at a subsidized price, and CHWs will be able to keep most or all of their sales. This is expected to encourage CHWs to attend more *Stratégie Avancée* and to spend more of their free time offering methods in their communities.

This study is the first to explore the acceptability of the provision of DMPA-SC by CHWs in a rural population of the DRC, and it adds to the growing body of literature that suggests DMPA-SC may be a “game changer” for task-shifting FP services in high-need areas. Women in the study population found DMPA-SC acceptable as a method and CHW provision satisfying as a distribution mechanism. Despite some limitations, including use of a convenience sample, this pilot represents an important step in obtaining official MOH authorization for the scale-up of this mechanism of distribution to other underserved regions in the DRC. Expanding contraceptive method choice in hard-to-reach areas through innovative practices may have a positive impact on contraceptive use and unmet need.

## Footnotes

1 Selection criteria included active CHW status for more than 2 years, at least some education, residence in the study area, having interest in learning how to inject DMPA-SC and willingness to work on a voluntary basis.
